# Increased risk of miscarriage among women experiencing physical or sexual intimate partner violence during pregnancy in Guatemala City, Guatemala: cross-sectional study

**DOI:** 10.1186/1471-2393-11-49

**Published:** 2011-07-06

**Authors:** Mira Johri, Rosa E Morales, Jean-François Boivin, Blanca E Samayoa, Jeffrey S Hoch, Carlos F Grazioso, Ingrid J Barrios Matta, Cécile Sommen, Eva L Baide Diaz, Hector R Fong, Eduardo G Arathoon

**Affiliations:** 1 Unité de Santé Internationale (USI), Centre de Recherche du Centre Hospitalier de l'Université de Montréal(CRCHUM), Montreal, Canada; 2 Department of Health Administration, University of Montreal, Montreal, Canada; 3 Asociación de Salud Integral (ASI), Guatemala City, Guatemala; 4 Clínica Familiar Luis Ángel García (CFLAG), Guatemala City, Guatemala; 5 Departments of Epidemiology, Biostatistics, and Occupational Health, McGill University, Montreal, Canada; 6 Centre for Clinical Epidemiology and Community Health, Lady Davis Institute for Medical Research, Montreal, Canada; 7 Centre for Research on Inner City Health, St Michael's Hospital, Toronto, Canada; 8 Department of Health Policy, Management and Evaluation, University of Toronto, Toronto, Canada; 9 Department of Paediatrics, Hospital General San Juan de Dios, Guatemala City, Guatemala; 10 Department of Gynaecology and Obstetrics, Hospital General San Juan de Dios, Guatemala City, Guatemala; 11 Observatoire Régional de Santé d'île de France, Paris, France; 12 Executive Director, Hospital General San Juan de Dios, Guatemala City, Guatemala

## Abstract

**Background:**

Violence against women by their male intimate partners (IPV) during pregnancy may lead to negative pregnancy outcomes. We examined the role of IPV as a potential risk factor for miscarriage in Guatemala. Our objectives were: (1) To describe the magnitude and pattern of verbal, physical and sexual violence by male intimate partners in the last 12 months (IPV) in a sample of pregnant Guatemalans; (2) To evaluate the influence of physical or sexual IPV on miscarriage as a pregnancy outcome.

**Methods:**

All pregnant women reporting to the maternity of a major tertiary care public hospital in Guatemala City from June 1st to September 30th, 2006 were invited to participate in this cross-sectional study. The admitting physician assessed occurrence of miscarriage, defined as involuntary pregnancy loss up to and including 28 weeks gestation. Data on IPV, social and demographic characteristics, risk behaviours, and medical history were collected by interviewer-administered questionnaire. Laboratory testing was performed for HIV and syphilis. The relationship between IPV and miscarriage was assessed through multivariable logistic regression.

**Results:**

IPV affected 18% of the 1897 pregnant Guatemalan women aged 15-47 in this sample. Verbal IPV was most common (16%), followed by physical (10%) and sexual (3%) victimisation. Different forms of IPV were often co-prevalent. Miscarriage was experienced by 10% of the sample (*n *= 190). After adjustment for potentially confounding factors, physical or sexual victimisation by a male intimate partner in the last 12 months was significantly associated with miscarriage (ORadj 1.1 to 2.8). Results were robust under a range of analytic assumptions.

**Conclusions:**

Physical and sexual IPV is associated with miscarriage in this Guatemalan facility-based sample. Results cohere well with findings from population-based surveys. IPV should be recognised as a potential cause of miscarriage. Reproductive health services should be used to screen for spousal violence and link to assistance.

## Background

Violence against women by their male intimate partners (IPV) has a direct impact on the survival and quality of life of women and children worldwide. It is increasingly recognised as an important determinant of women's health and well being and, when it occurs during pregnancy, of poor birth outcomes [1-3]. Recently, attention has also turned to a possible link between violence during pregnancy and higher rates of non-birth outcomes such as unwanted pregnancy, miscarriage, and induced abortion [4-11].

Miscarriage is the most common negative gestational outcome occurring in about 20% of clinically recognised pregnancies [12]. Although chromosomal abnormalities may account for a substantial share of cases, loss related to chromosomal abnormality becomes less prevalent as gestational age increases. Miscarriage is a heterogeneous term and mechanisms for first trimester pregnancy loss likely differ from mechanisms for losses occurring later in gestation. Other determinants of involuntary early pregnancy loss are not yet well understood [13]. General studies of the determinants of miscarriage have typically not considered IPV [13].

Evidence concerning the role of IPV as a risk factor for miscarriage is currently ambiguous. A single, prospective case-control study of physical violence during pregnancy and miscarriage risk found no association [9]. Observational studies that do not control for confounding, case reports and qualitative studies suggest a possible link [10,14,15]. Several important studies using standardised population based cross-sectional household surveys report positive associations [2,4-6,8,11,16]. However, findings from household surveys suffer from numerous limitations. In general, reports of IPV and miscarriage are for lifetime occurrence and it is impossible to establish a temporal sequence. The direction of causality is uncertain in that occurrence of miscarriage may also provoke partner violence. Information on exposures and outcomes is provided by survey respondents who may recall events imperfectly and have difficulty in distinguishing consistently between miscarriage, abortion and stillbirth [16]. Standardised survey data contains limited information to control for important possible confounders.

Guatemala is a lower middle income Central American nation of 13.4 million inhabitants with a generally high prevalence of IPV, high fertility rates, limited access to health care for the poor, and considerable social and gender inequality [17-19]. We analysed data from a hospital-based sample of pregnant women in Guatemala City to clarify the relationship between IPV and miscarriage. Our objectives were: (1) to describe the magnitude and pattern of different types of IPV experienced by women in this sample, and to identify risk factors for these conditions; and (2) to evaluate the influence of verbal, physical and sexual abuse by male intimate partners in the last 12 months on miscarriage. Based on our review of the literature and our conceptual model (see Methods), we hypothesised that physical and sexual abuse would be causally related to higher rates of miscarriage but that verbal abuse would not be so related.

## Methods

### Study design

A cross-sectional study of pregnant women reporting to the maternity ward at the Hospital General San Juan de Dios (HGSJD), Guatemala City during 2006.

### Setting

Data were collected via a project to reduce mother-to-child transmission (MTCT) of HIV at the Hospital General San Juan de Dios (HGSJD), one of Guatemala's two national hospitals. Located in Guatemala City, the HGSJD is a major teaching hospital serving a poor and ethnically diverse patient population. The original goals of the HIV MTCT study were (1) to determine HIV prevalence in pregnant women at this site; and (2) to evaluate the impact of the HIV MTCT programme on clinical outcomes. To meet these objectives, the study collected extensive data on experiences of gender-based violence and pregnancy outcomes. Methods are described in detail elsewhere [20].

Briefly, the MTCT study was conducted in two phases. Intake for the first phase took place in antenatal care at the HGSJD in 2005. Recruitment for the second phase, from which data for this analysis are drawn, took place in the HGSJD maternity in 2006. The HGSJD maternity includes a birthing centre and admissions for pregnancy-related emergencies. All pregnant women reporting to the facility were offered counselling and an HIV test. Hepatitis B and syphilis testing were also performed for women reporting to the maternity ward. Patients testing positive for HIV, hepatitis B or syphilis received treatment for their conditions. Those reporting IPV victimisation were referred to a hospital counsellor.

An independent Guatemalan ethics committee (Comité de Ética Independiente Zugueme) certified by the US National Institutes of Health approved study protocols. All participants offered written informed consent.

### Participants

All pregnant women reporting to the maternity ward of the HGSJD during the intake period were eligible to participate. Study intake ran from 2006-06-01 to 2006-09-30, 7 days per week during daytime hours. Women reported for a variety of indications, including labour, pregnancy complications and miscarriage. We excluded women who did not provide informed consent.[Additional file 1, Figure S1]

### Data sources and sample size

The principal data source was an interviewer-administered questionnaire collecting socio-demographic data, a detailed medical history, and social and behavioural factors related to HIV and poor birth outcomes. For key variables, complementary diagnostic information was collected from physicians or through laboratory testing. All variables and measures were assessed during pregnancy.

Our study was designed to have 80% power to detect a minimum odds ratio of 2 for increased miscarriage risk in the exposed group with an alpha = 0.05. Based on assumptions of a 10% prevalence of physical or sexual IPV in the study population and a 15% miscarriage rate in the non-exposed group, our recruitment targets were 116 in the exposed group and 1147 in the non-exposed group, for a total sample size of 1263 using the method of Fleiss (with continuity correction) [21].

### Variables and measures

#### Outcome

A miscarriage is any pregnancy that ends unintentionally before the foetus is viable. Gestational age at viability varies among countries. In Guatemala, pregnancy loss up to and including 28 weeks gestation was identified as an appropriate standard. For each patient, we asked the admitting physician to specify a single reason for presentation to the maternity ward from a list comprising labour, false labour, miscarriage, complications of elective abortion, pregnancy complications, and other. Patients were considered to have had a miscarriage if the admitting physician selected miscarriage as the reason for presentation. Cases were crosschecked against gestational age to ensure consistency with study definitions.

#### Exposures

Questions on gender-based violence were adapted from the World Health Organization (WHO)'s Multicountry Study on Women's Health and Domestic Violence [1,2,22]. These questions have been incorporated into Demographic Health Surveys (DHS) worldwide, including Guatemala's National Survey of Maternal-Child Health (ENSMI) [17]. IPV was assessed through the following three questions: "During the past 12 months, has your partner: (1) Abused you verbally? E.g. said or done something to humiliate you? Insulted you or called you by offensive names? (2) Abused you physically? E.g. Hit, kicked or slapped you? Thrown an object at you? (3) Abused you sexually? E.g. obliged you to have sexual relations against your will?" IPV exposures were constructed as binary (yes, no) variables. Participants were recorded as experiencing verbal IPV if they answered "yes" to question 1, physical IPV if they answered "yes" to question 2, and sexual IPV if they answered "yes" to question 3.

#### Potential confounders or effect modifiers

Demographics (age, education, religion, civil status, ethnicity), childhood sexual abuse, pregnancy number and risk behaviours (use of tobacco in the last 6 months, use of alcohol in the last 6 months, lifetime use of illegal drugs) were assessed using questions drawn from the ENSMI [17]. Ethnic categories important in Guatemala include the indigenous (Mayan-descended) and ladino (a heterogeneous Spanish-speaking population that shares cultural traits of Hispanic origin and wears western clothing) populations.

Participants' economic status was classified through a list of assets including ownership of household goods and dwelling characteristics taken from the ENSMI [17]. Based on multiple correspondence analysis, we excluded one item (solar energy) from the list and summed the remaining 8 to create a relative index of household wealth ranging from 0 (poorest) to 8 (best off).

Syphilis was initially evaluated using the Determine Syphilis TP rapid test (Abbott Diagnostics, North Chicago, IL). Those testing positive also received the fluorescent treponemal antibody absorbed (FTA-ABS) test. Syphilis cases are patients with two positive tests.

### Bias

Analytic bias could occur if cases of induced abortion were counted as cases of spontaneous miscarriage. The choice to end a pregnancy has been linked to IPV in several previous studies [2,5,6,8,23]. Abortion is a prosecutable offence under Guatemalan law, making it impossible to identify cases through self-report. We asked the admitting physicians confidentially to identify probable cases of induced abortion for all admissions during the study period. Of the 12 cases identified, five reported IPV, four were less than 18 years of age, and five stated their civil status as "on own." Only one patient had none of these factors. None had a low wealth index score. These 12 cases were retained in the analysis in the group that did not experience a pregnancy outcome of miscarriage.

### Conceptual framework

**Figure 1:**
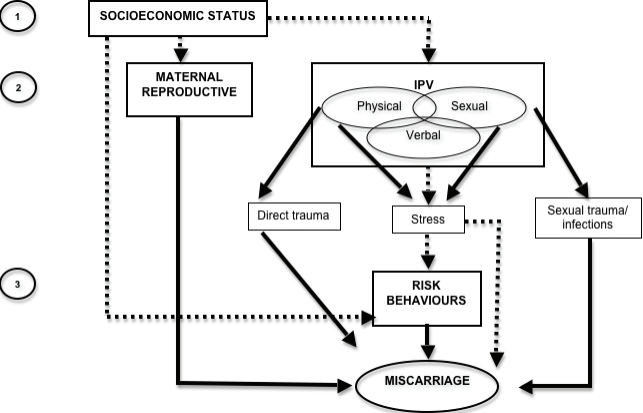
**Conceptual hierarchical framework describing mechanisms for the impact of IPV during pregnancy on miscarriage (adapted from Coker et al., 2004)**. Solid arrow denotes direct causal pathway. Dashed arrow denotes indirect causal pathway.

We adapted a conceptual framework describing the relationship between IPV and adverse pregnancy outcomes [24] to reflect the hierarchical relationships between risk factors contributing to miscarriage [25]. [Figure 1]

Level 1 considers socioeconomic determinants. In our model, distal factors linked to socioeconomic status (SES) have a potential effect on miscarriage via their influence on more proximate determinants. For example, education and wealth may influence maternal reproductive factors such as age at pregnancy or number of children. In Guatemala, low SES may also affect access to care for conditions such as sexually transmitted infections. SES also influences IPV by shaping options for choice of partner and social and economic barriers to exit from abusive relationships. SES is represented through the variables wealth, education and ethnicity.

Level 2 considers maternal reproductive factors and IPV, which are conceived as functioning largely independently and in parallel. Maternal reproductive factors can lead directly to miscarriage. We consider maternal age (a proxy for chromosomal abnormality linked to higher miscarriage risk), pregnancy number (since first pregnancies have higher miscarriage risk), and presence of active syphilis (a known contributor to miscarriage) [13,26].

Following Coker and colleagues [24], we postulated that physical IPV could lead to miscarriage through a direct mechanism consisting of physical trauma leading to pregnancy complications,[15] or an indirect mechanism involving increased stress, potentially affecting immunologic or endocrine functions, exacerbating pre-existing chronic illnesses, and increasing maternal risk behaviours [7,27]. Sexual IPV could also lead to miscarriage through similar indirect mechanisms, or directly through sexual trauma and infections leading to foetal loss [26,28-30]. Verbal IPV involves only an indirect route.

Level 3 considers three maternal risk behaviours: use of alcohol, tobacco or illegal drugs. These risk behaviours may lead directly to miscarriage, [31-35] and are conceived as lying to some degree on the causal pathway from IPV to miscarriage [3]. To the extent that they do fall on the causal pathway one should not adjust for their effect.

### Statistical methods

We used univariate and bivariate analyses to describe the magnitude and pattern of abuse in the sample and bivariate analysis to identify risk factors for abuse. Crude associations were assessed using univariable logistic regression for continuous variables and the χ2 test for categorical variables. Categorical variables with expected cell counts of 5 or less were examined via Fisher's exact test.

#### Main analysis

We used multivariable logistic regression to analyse the relationship between IPV and miscarriage, following an approach suggested by Hosmer and Lemeshow [36]. Possible causal relationships were outlined based on our conceptual model. Additional variables were identified through bivariate analyses describing risk factors for miscarriage. Those significant at a threshold of *p *= 0.25 and judged plausible as causal factors or proxies were included. Regression analyses were restricted to individuals with complete data on all variables. Additional analyses explored whether cases with missing data were related to any observed variables [37].

Modelling for the main analysis began with a model including only the exposure (physical or sexual abuse) and outcome (miscarriage) variables. We next entered variables in blocks corresponding to our conceptual model [38]. For Model 1, we entered all variables related to SES and an additional variable (occupation housewife) identified through bivariate exploration. We eliminated variables one at a time according to lack of significance at the *p *= 0.05 level and examined the change in coefficient size for the exposure variable due to removal of a potential confounder or effect modifier. If the change was greater than 10% the variable was retained even when the *p *value was larger than 5%; otherwise, it was removed [39]. For categorical variables with several levels we used the likelihood ratio test to study the change in the log likelihood resulting from removal of the group of associated dummy variables. If the test was not significant we removed the dummy variables [36]. To the model resulting from this iterative procedure, Model II introduced conceptual framework variables related to maternal reproductive factors and an additional variable (pregnancy planned) and repeated the previous modelling procedure. Model III added variables related to maternal risk behaviours and repeated the modelling procedure.

For continuous variables we examined the assumption of a linear relationship to the log odds of the outcome using lowess smoothers, local mean regression and inspection of quartiles. We used a linear association to model wealth, a cubic spline with three knots to model pregnancy number, and a categorical variable using ENSMI age categories to model age [17]. We also modelled age as a cubic spline with four knots. Regression results were qualitatively identical.

We investigated possible interactions between the exposure variable and plausible confounders. Models with and without interactions were compared using the likelihood ratio test. We fit the final model 1000 times on a bootstrap sample equal in size to the original sample to obtain more robust confidence intervals.

#### Supplementary Analyses

In supplementary analyses, we used the same procedures to consider physical, sexual and verbal abuse as independent exposure variables. To reduce heterogeneity in the outcome variable, we also used univariable logistic regression to study the relationship between all forms of IPV and miscarriage timing: "early" (before 13 weeks) versus "late" (from 13 to 28 weeks).

Analyses were performed using Stata 11 [40].

## Results

### Participants

Of the 2072 women eligible for this study, 8 (0.4%) were excluded due to failure to consent. An additional 167 (8.1%) were excluded due to missing data. The most common missing values were the diagnosis code indicating reason for presenting to the maternity ward (n = 110, 5.3%) followed by responses to the IPV variables (n = 56, 2.7%). All other variables were missing with less than 1% frequency. Missing variables were not related in regression analyses to any observed variables. A total of 1897 women (91.6% of those eligible) were included in the analysis [Additional file 1, Figure S1]. For the 1897 women included in the study, the following reasons for presenting to the HGSJD maternity were recorded by the admitting physicians: labour (*n *= 783, 41.28%), pregnancy complications (*n *= 581, 30.63%), false labour (*n *= 228, 12.02%), miscarriage (*n *= 194, 10.23%), risk of miscarriage, (*n *= 99, 5.22%), and induced abortion (*n *= 12, 0.63% (12 women)). Four cases originally diagnosed as miscarriage occurred later than 28 weeks gestation and were reclassified.

### Prevalence and co-prevalence of forms of IPV

**Figure 2:**
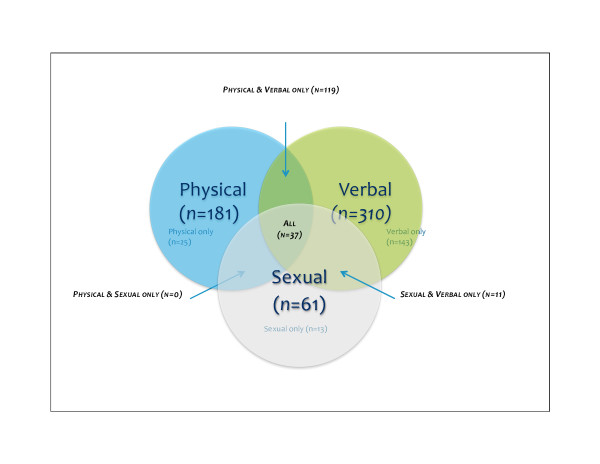
**Prevalence of intimate partner violence for 1897 pregnant women in Guatemala City, 2006**. Venn diagram describing relationships between the three forms of IPV considered in this study. 348 of 1897 women (18%) reported experiencing one or more forms of IPV in the last 12 months. Numbers are counts of positive responses.

Victimisation by a male intimate partner in the last 12 months was reported by 348 (18%) of the 1897 women in this study.[Figure 2]. Verbal abuse was reported by 310 (16%), physical abuse by 181 (10%), and sexual abuse by 61 (3%) of respondents. Different forms of IPV often occurred together. Of the 310 women reporting verbal IPV, 167 (54%) reported another form of abuse. Of the 181 women experiencing physical IPV, 156 (86%) also experienced another form of abuse, most commonly verbal (119 of 181, 66%). Of the 61 women experiencing sexual IPV, 48 (79%) experienced other forms of abuse, most commonly all three forms (37 of 61, 61%).

### Demographic and health characteristics related to IPV victimisation

Table 1 provides information on demographic and health characteristics and associations with IPV victimisation. IPV was not associated with ethnicity. Women experiencing physical IPV more frequently had low levels of wealth; no other form of IPV was associated with wealth. Women reporting verbal abuse, sexual abuse and any form of abuse were slightly older than the sample average. Having no education appeared to be protective for verbal IPV and any IPV; however higher rates of any, physical and sexual IPV were reported for those with only a primary education. No associations were seen at higher levels of education. Married women reported lower rates and those living on their own reported higher rates of IPV. Only 36% of the women in this sample planned their pregnancy; rates were lower for any IPV, physical IPV and verbal IPV. IPV in all forms was strongly related to childhood sexual abuse and use of alcohol, tobacco, and illegal drugs. Only 12 cases of syphilis were identified and no associations were seen with IPV. Miscarriage was experienced by 190 (10%) of the women in this sample and was associated with all forms of IPV. The 1549 women reporting no IPV had 143 cases of miscarriage (9%). The 348 women reporting one or more forms of IPV experienced 47 cases of miscarriage (14%).

**Table 1:** Demographic and health characteristics and experiences of IPV in the last year among 1897 pregnant women in Guatemala City, 2006^1, 2^

	All *(n = 1897)* *n *(%)	Any IPV *(n = 348)* *n *(%)	Physical IPV *(n = 181)* *n *(%)	Sexual IPV *(n = 61)* *n *(%)	Verbal IPV *(n = 310)* *n *(%)
**Age**					
**15-19**	437 (23)	73 (21)	34 (19)	11 (18)	68 (22)
**20-24**	538 (28)	90 (26)	60 (33)	13 (21)	76 (25)
**25-29**	413 (22)	74 (21)	34 (19)	9 (15)	68 (22)
**30-34**	280 (15)	60 (17)	34 (19)	16 (26)**	55 (18)
**35-39**	148 (8)	38 (11)*	14 (8)	9 (15)*	33 (11)*
**40-49**	70 (4)	10 (0)	4 (2)	2 (3)	9 (3)
**Indigenous ethnicity**^3^	329 (17)	52 (15)	33 (18)	13 (21)	48 (16)
**Religion**					
**Catholic**	961 (51)	162 (47)	86 (48)	33 (54)	139 (45)*
**Evangelical**	666 (35)	119 (34)	56 (31)	18 (30)	108 (35)
**Other/none**	269 (14)	67 (19)**	39 (22)**	10 (16)	63 (20)***
**Civil Status**					
**Married**	557 (29)	74 (21)***	40 (22)*	12 (20)	63 (20)***
**Living together**	1046 (55)	192 (55)	92 (51)	27 (44)	181 (58)
**On own**	294 (16)	82 (24)***	49 (27)***	22 (36)***	66 (21)**
**Wealth Index**^4^					
**Low**	274 (14)	60 (17)	35 (19)*	8 (13)	55 (18)
**Medium**	930 (49)	165 (47)	88 (49)	34 (56)	142 (46)
**High**	693 (37)	123 (35)	58 (32)	19 (31)	113 (37)
**Education**^5^					
**None**	287 (15)	37 (11)**	21 (12)	8 (13)	32 (10)**
**Primary**	1012 (53)	204 (59)*	109 (60)*	40 (66)*	178 (57)
**Secondary**	513 (27)	93 (27)	47 (26)	10 (16)	86 (28)
**Some College**	85 (5)	14 (4)	4 (2)	3 (5)	14 (5)
**Occupation housewife**	1542 (81)	256 (74) ***	129 (71)***	42 (69)**	228 (74)***
**Sexual abuse age 12 or less**	181 (10)	61 (18)***	40 (22)***	16 (26)***	56 (18)***
**Pregnancy planned**	683 (36)	93 (27)***	51 (28)*	17 (28)	84 (27)***
**Risk behaviours^6^**					
**Alcohol**	93 (5)	35 (10)***	19 (11)***	9 (15)***	32 (10)***
**Tobacco**	50 (3)	20 (6)***	13 (7)***	6 (10)***	17 (6)***
**Illegal drugs**	33 (2)	12 (4)**	9 (5)***	5 (8)***	11 (4)**
**Syphilis**^7^	12 (1)	2 (1)	1 (1)	2 (3)	1 (0)
**Miscarriage**^8^	190 (10)	47 (14)*	28 (16)**	12 (20)*	44 (14)**

^1^Statistically significant differences denoted as: * *p *< = 0.05, ** *p *< = 0.01, *** *p *< = 0.001

^2^Characteristics of the total sample are presented in the column labelled "All". For a given row variable, *p-*values refer to a comparison between the group designated in the column heading and its complement. For example, the column "Any IPV" compares those responding "yes" either to physical IPV or sexual IPV or verbal IPV versus those responding "no" to all of these questions. The column "Physical IPV" compares those responding "yes" to physical IPV versus those responding "no" to physical IPV.

^3^Ethnicity was self-identified; the rest of the sample identified as ladina

^4^An index of household goods ranging from 0-8. For purposes of presentation, we classified scores between 0-4 as low, 5-6 as medium, and 7-8 as high.

^5^Highest level of education completed.

^6^Consumption of alcohol or tobacco in the last 6 months, or lifetime consumption of illegal drugs.

^7^Syphilis cases were laboratory confirmed.

^8^The admitting physician assessed miscarriage.

### Main analysis: association of physical or sexual IPV to miscarriage

Table 2 presents regression results describing the association of physical or sexual IPV to miscarriage. The crude odds ratio (OR) shows that women reporting physical or sexual IPV during the 12 months prior are more likely to experience a miscarriage (OR = 1.88, 95% CI: 1.25 to 2.82, *p *= 0.002). Model I adjusted for SES; no variables in this group were significant. Of the factors related to maternal reproduction considered in Model II, only age was significant and retained in the model. The adjusted OR for Model II shows that women reporting physical or sexual IPV during the 12 months prior are more likely to experience a miscarriage (OR = 1.83, 95% CI: 1.23 to 2.72, *p *= 0.003). Of the behavioural risks considered in Model III, tobacco use was an important predictor of miscarriage. The adjusted OR for Model III shows that women reporting physical or sexual IPV during the 12 months prior are more likely to experience a miscarriage (OR = 1.69, 95% CI: 1.13 to 2.57, *p *= 0.014). An interaction term between tobacco use and the exposure was not significant.

**Table 2:** Associations between physical or sexual IPV in the last year and miscarriage as a pregnancy outcome in a sample of 1897 Guatemalan women ages 15-49^1, 2^

Variable	Crude OR (95% CI)	**Model I**^3^ OR (95% CI)	**Model II**^4^ OR (95% CI)	**Model III**^5^ OR (95% CI)
**Physical or sexual IPV**	1.88 (1.25 to 2.82) *p *= 0.002	1.88 (1.26 to 2.79) *p *= 0.002	1.83 (1.23 to 2.72) *p *= 0.003	1.69 (1.13 to 2.57) *p *= 0.014
**Age**				
**15-19**			1.0	1.0
**20-24**			1.89 (1.16 to 3.07) *p *= 0.010	1.85 (1.14 to 3.01) *p *= 0.013
**25-29**			1.45 (.85 to 2.46) *p *= 0.171	1.44 (.84 to 2.45) *p *= 0.184
**30-34**			1.88 (1.10 to 3.23) *p *= 0.022	1.91 (1.11 to 3.29) *p *= 0.019
**35-39**			3.10 (1.74 to 5.53) *p *< 0.001	3.08 (1.72 to 5.51) *p *< 0.001
**40-49**			3.59 (1.75 to 7.37) *p *< 0.001	3.76 (1.83 to 7.70) *p *< 0.001
**Tobacco**^6^				3.39 (1.72 to 6.68) *p *< 0.001

^1^Statistically significant differences denoted as: * *p *< = 0.05, ** *p *< = 0.01, *** *p *< = 0.001

^2^Models I-III present confidence intervals and p-values based on bootstrap replications (n = 1000).

^3^Model I adjusted for physical or sexual IPV, and for four variables related to socioeconomic status: ethnicity, education, wealth, and occupation housewife. None of the factors related to SES were significant at the *p *< = 0.05 level.

^4^Model II adjusted for Model I variables and 4 additional maternal reproductive variables: maternal age, pregnancy number, presence of syphilis, and pregnancy planned. Syphilis could not be used due to collinearity. Maternal age was significant at the *p *< = 0.05 level.

^5^Model III adjusted for Model II variables and three additional risk behaviours: tobacco use during the last 6 months, alcohol use during the last 6 months, and use of illegal drugs (ever). Tobacco was significant at the *p *< = 0.05 level.

^6^Consumption of tobacco in the last 6 months.

### Supplementary analyses

#### Alternative exposure variables

Additional files 2, 3 and 4 (Tables S1, S2 and S3) present multivariable regression results for three analyses considering alternative exposure variables. Results for analyses considering physical IPV [Additional file 2, Table S1] and verbal IPV [Additional file 4, Table S3] as independent exposures were qualitatively identical to those from the main analysis. Results for a model considering sexual IPV [Additional file 3, Table S2] as an independent exposure were qualitatively identical for Models I and II; however, sexual IPV was not statistically significant in Model III. Each analysis also considered an interaction term between tobacco use and the exposure; none were significant. Bivariate analysis revealed no association between miscarriage and verbal IPV for the 143 women who experienced only verbal abuse (χ^2 ^= 0.0087, *p *= 0.926).

#### Gestational age at miscarriage

Additional file 5, Table S4 presents results of an analysis exploring potential differences in causal mechanisms at early and late gestational ages. There were 131 early and 59 late miscarriages. Univariable logistic regression showed that, for each form of IPV, odds ratios describing the influence of IPV on miscarriage were higher for late miscarriages; however, the trend was not statistically significant (physical or sexual abuse and early miscarriage, OR 1.75, CI: 1.08 to 2.83, *p *= 0.024; physical or sexual abuse and late miscarriage OR 1.94, CI: 0.99 to 3.80; *p *= 0.053).

## Discussion

### Principal findings

Intimate partner violence affected almost one in five of the 1897 pregnant women in this Guatemalan facility-based sample. Verbal IPV was most common (16%), followed by physical (10%) and sexual (3%) victimisation. Different forms of IPV were often co-prevalent. After adjustment for potentially confounding factors, regression results showed that physical or sexual victimisation by an intimate partner in the last 12 months was significantly associated with miscarriage. These findings were very robust. Under a range of analytic assumptions reflecting alternative ways of conceiving causal and confounding relationships, those reporting physical or sexual IPV were more likely to experience a miscarriage as compared to those without these experiences of violence (OR 1.1 to 2.8). Three supplementary analyses modelling alternative forms of the exposure variable showed similar results.

### Strengths and weaknesses of the study

Particular strengths of this study include its high acceptance rate and the completeness of information collected, enabling over 90% of eligible individuals to be included in the analyses. Admitting physicians ascertained miscarriage at the time of occurrence permitting accuracy in timing of gestational loss and reducing the risk of recall bias or misreporting. The time period required for IPV recall was similarly short, reducing the risk of recall bias. Exposure status was unknown at the time the outcome was ascertained. Diagnosis of outcome by admitting physicians also helped to mitigate potential misclassification bias due to cases of induced abortion presenting as miscarriage. In addition, despite the fact that data were collected at a single time-point, the study design permits a clear assessment of temporal sequence. As patients were identified as having a miscarriage upon presentation to the maternity ward and then asked about experiences of IPV in the last 12 months, it is clear that IPV victimisation preceded the miscarriage. Moreover, the experience of IPV is likely concurrent with pregnancy. Adoption of a conceptual framework to aid in defining causal relationships and an appropriate statistical approach to control for confounding are also important assets. Sample size is adequate for this analysis as confidence intervals are sufficiently narrow to permit reporting of significant results. Together, these factors enhance the ability to infer from IPV as cause to miscarriage as effect, and thus, the study's internal validity.

The study also has several limitations:

(1) This study relies on self-report to assess exposure and confounding variables. Missing values occurred at low rates and women with missing values did not differ on observed variables from those included in the study; nonetheless, 3% of women declined to provide answers for the IPV variables. Self-reported rates of IPV are generally believed to be underestimates and refusal to answer may constitute evidence of IPV in this context, suggesting that our estimates of IPV prevalence are conservative.

(2) This is a hospital-based sample, raising questions about possible distortion of the exposure-disease relationship due to patient selection. The HGSJD is a large, public hospital catering to Guatemala City's uninsured, currently about 50% of the general population. The hospital manages both normal births and those with complications, of which pregnancy in women with pre-existing hypertension or diabetes figure as most important. Although pregnancies in this patient population are likely distinct from those in the general population, there is excellent evidence that exposure status does not influence patient selection. The distribution of the exposure variables is similar to nationally representative values. Our study population gave IPV prevalences of 16% verbal; 10% physical; and 3% sexual (*n *= 1897). Responses for physical and sexual IPV were virtually identical to those from the nationally representative ENSMI (25% verbal; 9% physical; 4% sexual IPV, (*n *= 6595) [17]. The lower prevalence of verbal abuse in our study is likely due to differences in question phrasing.

(3) Because many miscarriages do not require hospitalisation, this sample is truncated and would likely capture more serious cases. The clinical and epidemiological characteristics of miscarriage in a facility-based sample are likely to differ from those in a community-based sample, rendering applicability of findings to the general population uncertain. Notwithstanding, given our positive findings concerning the link between IPV and miscarriage, reliance on a clinical sample is unlikely to have biased the assessment of a causal relationship.

(4) We were unable to consider some potentially important confounding variables. Pre-pregnancy body mass index (BMI) has been correlated with miscarriage through very low BMI (under 18.5), possibly as a proxy for poor nutrition, [13] and high BMI (above 30) [41]. In the Phase I (antenatal care) sample from this study about 16% of patients fell into these BMI ranges, equally divided between very low and high categories.(Authors' unpublished findings) Due to logistical challenges, information on BMI was not routinely collected for the Phase II (maternity) sample analysed here. It is unclear whether BMI is systematically related to IPV victimisation; however, in the affirmative, BMI would plausibly lie on the causal pathway. Untreated sexually transmitted infections (STIs) such as syphilis, gonococcal infection, bacterial vaginosis and Chlamydia are documented causes of miscarriage [42]. With the exception of syphilis, information about these STIs was unavailable for our study. Nonetheless, STIs likely lie on the causal pathway from IPV to miscarriage as male partner infidelity is strongly correlated with IPV in Guatemala [18].

(5) We used physician judgment to identify probable cases of induced abortion at time of presentation to the hospital. Physician judgment is imperfect and there is no reliable means to assess what proportion of induced abortion cases were correctly identified. As anticipated, experiences of IPV were more common among women who induced abortion. Given this, misclassification of induced abortions as spontaneous abortions might spuriously strengthen the association between IPV and miscarriage. Due to its illegality in Guatemala, induced abortion is believed to be an infrequent reason for reporting to a public hospital. Impact on our results is therefore likely small, but in the absence of evidence the possibility of bias cannot be excluded.

(6) Our understanding of the factors causing miscarriage is incomplete and residual confounding remains a possibility.

### Relationship to other studies

Our results cohere well with those from several recent studies using household surveys to examine similar questions,[2,4-6,8,11,16] while permitting a clearer assessment of causality. By responding to unanswered questions in previous work concerning the temporal sequence of exposure and effect, proper control of confounding, and the influence of recall, measurement and misclassification bias, our study lends strength to the finding of a positive association between physical or sexual IPV victimisation and miscarriage. The finding that verbal abuse was also significantly related to miscarriage ran counter to our study hypothesis but was also found by Alio and colleagues [4]. We believe that this finding is likely a statistical artefact reflecting the co-prevalence of verbal and other forms of IPV. We found no association between the experience of verbal abuse alone and miscarriage.

Our findings contrast with a similar prospective case-control study from a United States ER that found no effect of physical violence on miscarriage [9]. Possible explanations include a different study population and context, and absence of a conceptual framework to motivate control of confounding variables coupled with a stepwise approach to statistical analysis. Notably, the study controlled for occurrence of a previous miscarriage [9], a variable likely to lie on the causal pathway [4].

### Interpretation

There is substantial evidence that physical and sexual IPV is associated with miscarriage in this Guatemalan facility-based sample. Similar results have been found across a variety of study designs and populations [2,4,5,8,11,16]. This association requires further assessment and replication to establish causality. At this juncture, there is insufficient evidence to support claims of a causal relationship between verbal IPV and miscarriage, despite the existence of plausible pathways from emotional and psychological abuse to physical outcomes [7,27].

Future research may help to clarify whether IPV is of greater importance for miscarriages occurring after the first trimester. Our findings on this point were inconclusive. Although odds ratios for IPV risk were higher for late as compared to early miscarriage, results were not statistically significant. This may reflect limited statistical power for this supplementary analysis related to the relatively small number of late occurring miscarriages.

We view substance use as part of the causal pathway leading from IPV exposure to miscarriage. We have presented odds ratios estimating the risk of miscarriage with (Model III) and without (Model II) controlling for risk behaviours related to use of alcohol, tobacco and illegal drugs because the degree to which these behaviours lie on the causal pathway is unknown and controversial. Both for Model II and Model III, IPV is a statistically significant risk factor for miscarriage. We believe that Model II is for statistical and conceptual reasons the best, suggesting an adjusted odds ratio for miscarriage risk of 1.2-2.7 for women experiencing physical or sexual IPV in our sample.

## Conclusions

Violence at the hands of male intimate partners is a routine occurrence for countless women in Guatemala and worldwide. Despite its damaging effects on the health and well being of women [1,2] there is a dearth of services for IPV victims in Guatemala. Help for abused children is almost non-existent, even with the mounting evidence that sexual and other forms of abuse are important risk factors for IPV and poor health outcomes across the life course [43]

Accumulating evidence from this study and others now demonstrates that the devastation of IPV may extend to unborn children [3-6,44]. Gender-based violence has traditionally been a low priority. Contact with health services during pregnancy offers a window of opportunity for redress. For the health of mother and child, it is imperative that IPV be recognised as a potential cause of miscarriage and that reproductive health services be used to screen for spousal violence and link to assistance. Other effective strategies for prevention of miscarriage also exist. Future research should quantify the local prevalence of untreated STIs to motivate selection of effective, context-specific interventions to prevent miscarriage and other adverse pregnancy outcomes.

## Competing interests

The authors declare that they have no conflicts of interest, financial or otherwise.

## Authors' contributions

MJ conceived and designed the study, analysed and interpreted the data, and drafted the manuscript. REM contributed to conception and design of the study, acquisition and interpretation of the data, and critical revision of the manuscript. JFB contributed to conception and design of the study, analysis and interpretation of the data, and critical revision of the manuscript. BS contributed to conception and design of the study, interpretation of the data, and critical revision of the manuscript. JSH contributed to conception and design of the study, data analysis, and critical revision of the manuscript. CFG contributed to conception and design of the study, interpretation of the data, and critical revision of the manuscript. JBM contributed to conception and design of the study, acquisition and interpretation of the data, and critical revision of the manuscript. CS contributed to data analysis and critical revision of the manuscript. ELBD contributed to acquisition of the data and critical revision of the manuscript. HF contributed to acquisition of the data and critical revision of the manuscript. EGA contributed to conception and design of the study, interpretation of the data, and critical revision of the manuscript.

All authors have approved the final manuscript version.

## Pre-publication history

The pre-publication history for this paper can be accessed here:

http://www.biomedcentral.com/1471-2393/11/49/prepub

## Supplementary Material

Additional file 1 **Figure S1. Analysis sample (*n *= 1897)**. Flow diagram depicting eligibility, inclusion and exclusion criteria used to define the analysis sample. ^1^Study intake ran from 2006-06-01 to 2006-09-30, seven days per week during daytime hours. ^2^Missing values for diagnosis of outcome and abuse were not related in regression analyses to any observed variables. Click here for file

Additional file 2 **Table S1. Associations between physical IPV in the last year and miscarriage as a pregnancy outcome in a sample of 1897 Guatemalan women ages 15-49^1, 2^**. Results of supplementary analysis considering the impact of physical IPV on miscarriage. ^1^Statistically significant differences denoted as: * *p *< = 0.05, ** *p *< = 0.01, *** *p *< = 0.001. ^2^Models I-III present confidence intervals and p-values based on bootstrap replications (n = 1000). ^3^Model I adjusted for physical or sexual IPV, and for four variables related to socioeconomic status: ethnicity, education, wealth, and occupation housewife. None of the factors related to SES were significant at the *p *< = 0.05 level. ^4^Model II adjusted for Model I variables and 4 additional maternal reproductive variables: maternal age, pregnancy number, presence of syphilis, and pregnancy planned. Syphilis could not be used due to perfect correlation with the outcome (all cases occurred in the "no miscarriage" group). Maternal age was significant at the *p *< = 0.05 level. ^5^Model III adjusted for Model II variables and three additional risk behaviours: tobacco use during the last 6 months, alcohol use during the last 6 months, and use of illegal drugs (ever). Tobacco was significant at the *p *< = 0.05 level. ^6^Consumption of tobacco in the last 6 months. Click here for file

Additional file 3 **Table S2. Associations between sexual IPV in the last year and miscarriage as a pregnancy outcome in a sample of 1897 Guatemalan women ages 15-49^1, 2^**. Results of supplementary analysis considering the impact of sexual IPV on miscarriage. ^1 ^Statistically significant differences denoted as: * *p *< = 0.05, ** *p *< = 0.01, *** *p *< = 0.001. ^2^Models I-III present confidence intervals and p-values based on bootstrap replications (n = 1000). ^3^Model I adjusted for physical or sexual IPV, and for four variables related to socioeconomic status: ethnicity, education, wealth, and occupation housewife. None of the factors related to SES were significant at the *p *< = 0.05 level. ^4^Model II adjusted for Model I variables and 4 additional maternal reproductive variables: maternal age, pregnancy number, presence of syphilis, and pregnancy planned. Syphilis could not be used due to perfect correlation with the outcome (all cases occurred in the "no miscarriage" group). Maternal age was significant at the p < = 0.05 level. ^5^Model III adjusted for Model II variables and three additional risk behaviours: tobacco use during the last 6 months, alcohol use during the last 6 months, and use of illegal drugs (ever). Tobacco was significant at the p < = 0.05 level. ^6^Consumption of tobacco in the last 6 months. Click here for file

Additional file 4 **Table S3. Associations between verbal IPV in the last year and miscarriage as a pregnancy outcome in a sample of 1897 Guatemalan women ages 15-49^1, 2^**. Results of supplementary analysis considering the impact of physical IPV on miscarriage. ^1^Statistically significant differences denoted as: * p < = 0.05, ** p < = 0.01, *** p < = 0.001. ^2^Models I-III present confidence intervals and p-values based on bootstrap replications (n = 1000). ^3^Model I adjusted for physical or sexual IPV, and for four variables related to socioeconomic status: ethnicity, education, wealth, and occupation housewife. None of the factors related to SES were significant at the p < = 0.05 level. ^4^Model II adjusted for Model I variables and 4 additional maternal reproductive variables: maternal age, pregnancy number, presence of syphilis, and pregnancy planned. Syphilis could not be used due to perfect correlation with the outcome (all cases occurred in the "no miscarriage" group). Maternal age was significant at the *p *< = 0.05 level. ^5^Model III adjusted for Model II variables and three additional risk behaviours: tobacco use during the last 6 months, alcohol use during the last 6 months, and use of illegal drugs (ever). Tobacco was significant at the *p *< = 0.05 level. ^6 ^Consumption of tobacco in the last 6 months. Click here for file

Additional file 5 **Table S4. Intimate partner violence (IPV) in the last year and "early"^1 ^versus "late"^2 ^miscarriage in a sample of 1897 Guatemalan women ages 15-49^3, 4^**. Results of supplementary analysis considering the impact of IPV on miscarriage, stratified by gestational age at occurrence of miscarriage (early vs. late). ^1^"Early" miscarriages are those occurring before 13 weeks gestation. ^2^"Late" miscarriages occurred from 13 to 28 weeks gestation, inclusive. ^3^This is an exploratory analysis using univariable logistic regression. Statistically significant differences are denoted as: * *p *< = 0.05, ** *p *< = 0.01, *** *p *< = 0.001. ^4^Results were confirmed using the χ^2 ^test, or Fisher's exact test (for cells with counts ≤ 5). No meaningful differences were found. Click here for file
