# Supplementary material for: Increased risk of miscarriage among women experiencing physical or sexual intimate partner violence during pregnancy in Guatemala City, Guatemala: cross-sectional study
Source: BMC Pregnancy Childbirth. 2011 Jul 6;11:49. doi: 10.1186/1471-2393-11-49 (PMC3150323; doi:10.1186/1471-2393-11-49)
Supplement: Additional file 1 — Figure S1. Analysis sample (n = 1897). Flow diagram depicting eligibility, inclusion and exclusion criteria used to define the analysis sample. 1Study intake ran from 2006-06-01 to 2006-09-30, seven days per week during daytime hours. 2Missing values for diagnosis of outcome and abuse were not related in regression analyses to any observed variables. [file 1471-2393-11-49-S1.DOC]

**Figure S1. Analysis sample (*n*=1897)**

Eligible to participate (*n* = 2072)

- Pregnant women reporting to the obstetrics & gynaecology emergency / delivery room at the HGSJD during the intake period^1^

Failure to consent (*n* = 8)

1 individual did not consent

7 consented but left prior to HIV rapid testing

Included in the study (*n* = 2064)

- All those eligible who gave consent (99.6% of 2072)

Missing values (*n* = 167)

Diagnosis code indicating reason for presenting (*n*=110; 5.3% of 2072)^2^

Responses to abuse variables (*n=*56; 2.7% of 2072)^2^

All other variables were missing with less than 1% frequency.

Analysis sample (*n* = 1897)

- All those included in the study with complete information (91.6% of 2072; 91.9% of 2064)

^1^Study intake ran from 2006-06-01 to 2006-09-30, seven days per week during daytime hours.

^2^Missing values for diagnosis of outcome and abuse were not related in regression analyses to any observed variables.
