# Supplementary material for: Increased risk of miscarriage among women experiencing physical or sexual intimate partner violence during pregnancy in Guatemala City, Guatemala: cross-sectional study
Source: BMC Pregnancy Childbirth. 2011 Jul 6;11:49. doi: 10.1186/1471-2393-11-49 (PMC3150323; doi:10.1186/1471-2393-11-49)
Supplement: Additional file 4 — Table S3. Associations between verbal IPV in the last year and miscarriage as a pregnancy outcome in a sample of 1897 Guatemalan women ages 15-491, 2. Results of supplementary analysis considering the impact of physical IPV on miscarriage. 1Statistically significant differences denoted as: * p < = 0.05, ** p < = 0.01, *** p < = 0.001. 2Models I-III present confidence intervals and p-values based on bootstrap replications (n = 1000). 3Model I adjusted for physical or sexual IPV, and for four variables related to socioeconomic status: ethnicity, education, wealth, and occupation housewife. None of the factors related to SES were significant at the p < = 0.05 level. 4Model II adjusted for Model I variables and 4 additional maternal reproductive variables: maternal age, pregnancy number, presence of syphilis, and pregnancy planned. Syphilis could not be used due to perfect correlation with the outcome (all cases occurred in the "no miscarriage" group). Maternal age was significant at the p < = 0.05 level. 5Model III adjusted for Model II variables and three additional risk behaviours: tobacco use during the last 6 months, alcohol use during the last 6 months, and use of illegal drugs (ever). Tobacco was significant at the p < = 0.05 level. 6 Consumption of tobacco in the last 6 months. [file 1471-2393-11-49-S4.DOC]

Table S3. Associations between verbal IPV in the last year and miscarriage as a pregnancy outcome in a sample of 1897 Guatemalan women ages 15-49^1, 2^

| **Variable** | **Crude OR (95% CI)** | **Model I** ^3^  **OR (95% CI)** | **Model II** ^4^  **OR (95% CI)** | **Model III** ^5^  **OR (95% CI)** |
| --- | --- | --- | --- | --- |
| **Verbal IPV** | 1.63 (1.14 to 2.34)  *p*= 0.008 | 1.63 (1.14 to 2.35)  *p*=0.008 | 1.61 (1.11 to 2.33)  *p*=0.011 | 1.53 (1.04 to 2.24)  *p*=0.030 |
| **Age** |  |  |  |  |
| **15-19** |  |  | Reference category | Reference category |
| **20-24** |  |  | 1.94 (1.20 to 3.14)  *p*=0.007 | 1.89 (1.17 to 3.06)  *p*=0.010 |
| **25-29** |  |  | 1.44 (0.85 to 2.44)  *p*=0.179 | 1.43 (.84 to 2.43)  *p*=0.191 |
| **30-34** |  |  | 1.90 (1.11 to 3.25)  *p*=0.019 | 1.93 (1.13 to 3.31)  *p*= 0.017 |
| **35-39** |  |  | 3.07 (1.72 to 5.49)  *p*<0.001 | 3.04 (1.69 to 5.47)  *p*<0.001 |
| **40-49** |  |  | 3.62 (1.77 to 7.39)  *p*<0.001 | 3.79 (1.86 to 7.75)  *p*<0.001 |
| **Tobacco** ^6^ |  |  |  | 3.49 (1.76 to 6.90)  *p*<0.001 |

^1^ Statistically significant differences denoted as: * *p* <= 0.05, ** *p* <= 0.01, *** *p* <= 0.001

^2^Models I-III present confidence intervals and p-values based on bootstrap replications (n=1000).

^3^Model I adjusted for physical or sexual IPV, and for four variables related to socioeconomic status: ethnicity, education, wealth, and occupation housewife. None of the factors related to SES were significant at the *p*<=0.05 level.

^4^Model II adjusted for Model I variables and 4 additional maternal reproductive variables: maternal age, pregnancy number, presence of syphilis, and pregnancy planned. Syphilis could not be used due to collinearity. Maternal age was significant at the *p*<=0.05 level.

^5^Model III adjusted for Model II variables and three additional risk behaviours: tobacco use during the last 6 months, alcohol use during the last 6 months, and use of illegal drugs (ever). Tobacco was significant at the *p*<=0.05 level.

^6^ Consumption of tobacco in the last 6 months
