# Supplementary material for: Increased risk of miscarriage among women experiencing physical or sexual intimate partner violence during pregnancy in Guatemala City, Guatemala: cross-sectional study
Source: BMC Pregnancy Childbirth. 2011 Jul 6;11:49. doi: 10.1186/1471-2393-11-49 (PMC3150323; doi:10.1186/1471-2393-11-49)
Supplement: Additional file 5 — Table S4. Intimate partner violence (IPV) in the last year and "early"1 versus "late"2 miscarriage in a sample of 1897 Guatemalan women ages 15-493, 4. Results of supplementary analysis considering the impact of IPV on miscarriage, stratified by gestational age at occurrence of miscarriage (early vs. late). 1"Early" miscarriages are those occurring before 13 weeks gestation. 2"Late" miscarriages occurred from 13 to 28 weeks gestation, inclusive. 3This is an exploratory analysis using univariable logistic regression. Statistically significant differences are denoted as: * p < = 0.05, ** p < = 0.01, *** p < = 0.001. 4Results were confirmed using the χ2 test, or Fisher's exact test (for cells with counts ≤ 5). No meaningful differences were found. [file 1471-2393-11-49-S5.DOC]

Table S4. Intimate partner violence (IPV) in the last year and “early”^1^ versus “late”^2^ miscarriage in a sample of 1897 Guatemalan women ages 15-49^3, 4^

| **Form of IPV** |  | **Odds Ratio** | ***p*-value** | **95% Confidence Interval** | |
| --- | --- | --- | --- | --- | --- |
| **Any IPV (physical, sexual, or verbal)** | | | | | |
|  | “Early” | 1.42 | 0.105 | 0.93 | 2.16 |
|  | “Late” | 1.69 | 0.080 | 0.94 | 3.03 |
| **Physical or Sexual IPV** | | | | | |
|  | “Early” | 1.75 | 0.024* | 1.08 | 2.83 |
|  | “Late” | 1.94 | 0.053 | 0.99 | 3.80 |
| **Physical IPV** | | | | | |
|  | “Early” | 1.57 | 0.092 | 0.93 | 2.64 |
|  | “Late” | 2.00 | 0.053 | 0.99 | 4.00 |
| **Sexual IPV** | | | | | |
|  | “Early” | 2.10 | 0.057 | 0.98 | 4.52 |
|  | “Late” | 2.27 | 0.125 | 0.80 | 6.49 |
| **Verbal IPV** | | | | | |
|  | “Early” | 1.50 | 0.065 | 0.98 | 2.31 |
|  | “Late” | 1.78 | 0.058 | 0.98 | 3.25 |

^1^ “Early” miscarriages are those occurring before 13 weeks gestation.

^2^ “Late” miscarriages occurred from 13 to 28 weeks gestation, inclusive.

^3^ This is an exploratory analysis using univariable logistic regression. Statistically significant differences are denoted as: * *p* <= 0.05, ** *p* <= 0.01, *** *p* <= 0.001

^4^ Results were confirmed using the χ^2^ test, or Fisher’s exact test (for cells with counts
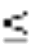
 5). No meaningful differences were found.
